# Matrine induces RIP3-dependent necroptosis in cholangiocarcinoma cells

**DOI:** 10.1038/cddiscovery.2016.96

**Published:** 2017-01-23

**Authors:** Beibei Xu, Minying Xu, Yuan Tian, Qiang Yu, Yujie Zhao, Xiong Chen, Panying Mi, Hanwei Cao, Bing Zhang, Gang Song, Yan-yan Zhan, Tianhui Hu

**Affiliations:** 1 Cancer Research Center, Xiamen University Medical College, Xiamen 361102, China; 2 Department of Basic Medicine, Xiamen University Medical College, Xiamen 361102, China

## Abstract

The development of acquired resistance to pro-apoptotic antitumor agents is a major impediment to the cure of cholangiocarcinoma (CCA). Antitumor drugs inducing non-apoptotic cell death are considered as a new approach to overcome such drug resistance. Here, we reported for the first time that matrine-induced necroptosis in CCA cell lines, differing from its classical role to induce apoptosis in many other kinds of cancer cells. CCA cells under matrine treatment exhibited typical necrosis-like but not apoptotic morphologic change. These matrine-induced morphologic change and cell death in CCA cells were greatly attenuated by necroptosis inhibitor necrostatin-1, but not apoptosis inhibitor z-VAD-fmk. Unlike many cancer cells with negative receptor-interacting protein 3 (RIP3) expression, moderate expression of RIP3 in CCA cells was observed and was required for matrine to induce necroptosis, which was switched to apoptosis after knocking down endogenous RIP3. Moreover, matrine could increase RIP3 expression level, which may facilitate the necroptosis process. Translocation of mixed lineage kinase-domain like (MLKL) from cytoplasm to plasma membrane as a downstream event of RIP3, as well as the increased production of reactive oxygen species (ROS) by RIP3/MLKL, was critical for matrine to induce necroptosis. In clinical study, we found RIP3 was lower but still moderately expressed in most CCA tissue samples compared with adjacent normal tissues. Taken together, we identified matrine as a necroptosis inducer in CCA by enhancing RIP3 expression and the following RIP3/MLKL/ROS signaling pathway, which provided new individualized strategies based on RIP3 expression to overcome chemoresistance in CCA therapy.

## Introduction

Cholangiocarcinoma (CCA) is one of the most common malignant tumors. Epidemiological data revealed that the incidence and mortality of CCA were increasing gradually during last three decades.^1^ Surgery is the most effective curative approach, however, only 10% of patients are suitable to accept surgery due to the difficulty of early diagnosis.^2,3^ Chemotherapeutics remains the chief therapeutic method for inoperable patients. Inducing apoptosis is one of the most important mechanisms of chemotherapeutic drugs to kill cancer cells. However, the development of acquired resistance to pro-apoptotic antitumor agents is a major obstacle in CCA chemotherapy.^3–5^ Hence, application of antitumor agents inducing non-apoptotic cell death may be a new way to overcome such drug resistance.

Necroptosis, characterized as the cell death with the similar morphology as necrosis and the unique signal pathway just as apoptosis, is also a kind of important programmed cell deaths.^6,7^ Necroptosis has caused wide public concern over the recent years owing to its important function in pathology and physiology, including developing tissue damage response, defending against viral infections, stimulating the immune system in response to infection, inhibiting or triggering inflammatory reactions.^8,9^ Many stimuli could induce necroptosis, one of which spurred by TNF-*α* is studied the most intensively.^10^ The receptor-interacting protein 3 (RIP3), a serine/threonine kinase in RIP kinase family, is necessary in TNF-*α*-induced necroptosis and has been identified as a key regulator in switching cell death from apoptosis to necrosis.^11^ However, RIP3 expression is silenced in most cancer cell lines. In addition, loss of RIP3 expression has also been observed in primary colon cancer tissue, primary breast cancer tissue, most acute myeloid leukemia samples and chronic lymphocytic leukemia.^12–14^ These reports indicate that RIP3 deficiency could be associated with cancer development and progression. As necroptosis is a new cell death pathway distinct from apoptosis, necroptosis inducer may bypass the apoptosis-resistant blockade in killing cancer cells,^15,16^ along with which the agents manipulating RIP3 expression will open the therapeutic possibility to make them sensitive to necroptosis.

In TNF-*α* induced necroptosis, TNF-*α* binding to TNF receptor 1 induces the formation of complex II, which contains RIP3, RIP1, Fas-associated protein with death domain and caspase-8.^17^ When caspase-8 inhibitors (for example, z-VAD-fmk) prevent caspase-8 activity or RIP3 expression is upregulated, RIP3 interacts with RIP1 through their RIP homotypic interaction motif domain to form a complex (called necrosome). After that, RIP3 is activated and initiate necroptosis via recruiting its downstream protein mixed lineage kinase-domain like (MLKL) and phosphorylating RIP1 and MLKL. Then MLKL translocates from cytoplasm to plasma membrane and participates in plasma membrane disruption through direct and indirect pathway.^18,19^

Matrine, an alkaloid isolated from traditional Chinese medicine *Sophora flavescens*, has been clinically used to treat various human diseases including inflammation, cardiac arrhythmias and hepatic fibrosis.^20,21^ Recently, matrine has drawn great attention owing to its antitumor effects. Accumulating evidence demonstrates that matrine suppresses cell proliferation via cell cycle arrest, inducing caspase-dependent and -independent apoptosis, and inhibiting migration and invasion in a variety of cancer cells.^22–24^ In the present study, a novel role of matrine, to induce necroptosis, was uncovered in CCA QBC939 and Mz-ChA-1 cell lines. The underlying mechanisms including why and how matrine induces necroptosis but not apoptosis in CCA were also demonstrated.

## Results

### Matrine-induced necroptosis but not apoptosis in CCA cells

**Figure 1:**
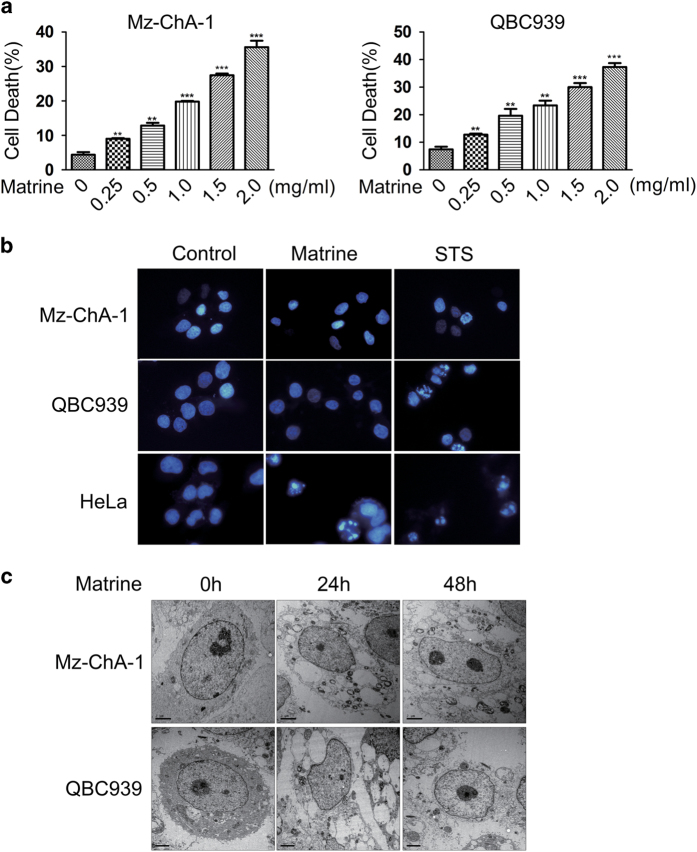
Matrine-induced non-apoptotic cell death in CCA cells. (**a**) Matrine-induced cell death in a dose-dependent manner in Mz-ChA-1 and QBC939 cells. Cells were treated with different concentrations (0, 0.25, 0.5, 1.0, 1.5 and 2.0 mg/ml) of matrine for 48 h, then the percentage of cell death was determined with PI staining plus flow cytometry. All data were presented as the mean±S.D. of three independent experiments. Significant differences compared with vehicle controls were indicated as *P<0.05, **P<0.01 and ***P<0.001 (assessed by Student’s *t*-test). (**b**) The nuclei of Mz-ChA-1 and QBC939 cells did not display typical apoptotic features under matrine treatment. Cells were treated with matrine (1.5 mg/ml) or vehicle (control) for 48 h, or STS (2 *μ*M) for 12 h, and then subjected to DAPI staining and fluorescent microscope. (**c**) Accurate morphological analysis of CCA cells treated with matrine using transmission electron microscope. Mz-ChA-1 and QBC939 cells were treated with matrine (1.5 mg/ml) for 24 and 48 h, and then observed and photographed under transmission electron microscope.

To investigate whether and how matrine induces cell death in CCA cells, we first employed flow cytometry to test the toxicity effect of matrine in Mz-ChA-1 and QBC939 cells. Results showed that matrine-induced cell death in a dose-dependent manner in these two cell lines (Figure 1a). Further morphology analysis using DAPI staining and fluorescence microscope showed that the nuclei of Mz-ChA-1 and QBC939 cells exhibited typical apoptotic features of hyper-condensation and fragmentation in the pro-apoptotic compound staurosporine-treated group, but not in the matrine-treated group (Figure 1b). However, the nuclei of HeLa cells displayed such typical apoptotic features in both the staurosporine-treated group and the matrine-treated group (Figure 1b). These results indicate that the cell death induced by matrine in CCA cells is highly unlikely to be apoptosis.

A more accurate morphological analysis using transmission electron microscope was performed to determine which kind of cell death was induced by matrine in CCA cells. Results showed that Mz-ChA-1 and QBC939 cells treated with matrine for 24 h presented extensive organelle and cell swelling and cytoplasmic vacuolation; these cells lost the plasma membrane integrity when treated with matrine for 48 h (Figure 1c). However, almost all the nuclei remained unchanged under matrine treatment (Figure 1c), consistent with the main characteristic of typical necrotic morphology. Thus, these results suggested that the cell death induced by matrine in CCA cells is very likely to be necrosis.

**Figure 2:**
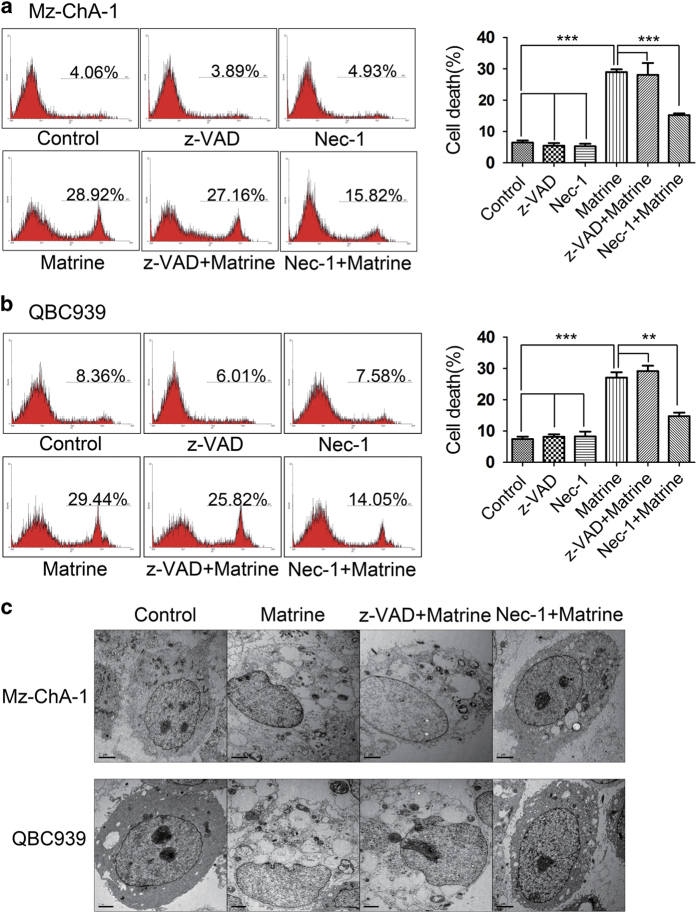
Matrine-induced necroptosis in CCA cells. (**a**–**c**) Mz-ChA-1 and QBC939 cells were pre-treated with necroptosis inhibitor Nec-1 (20 *μ*M) or caspase-dependent apoptosis inhibitor z-VAD-fmk (20 *μ*M) for 2 h, and then treated with matrine (1.5 mg/ml) or vehicle for 48 h. After that, the percentage of cell death was determined by PI staining and flow cytometry (**a** and **b**) and the accurate morphology of cells were observed and photographed under transmission electron microscope (**c**). Results were presented as the mean±S.D. from three independent experiments. Significant differences were indicated as *P<0.05, **P<0.01 and ***P<0.001 (assessed by Student’s *t*-test).

Necroptosis, a programmed form of necrosis, was reported to be induced by some compounds.^25–29^ To investigate whether matrine-induced cells death is necroptosis, Mz-ChA-1 and QBC939 cells were pre-treated with necroptosis inhibitor Nec-1 (RIP1 inhibitor) or apoptosis inhibitor z-VAD-fmk (pan-caspase inhibitor) before matrine treatment. The flow cytometry results showed that matrine-induced cell death was significantly blocked by Nec-1 but not z-VAD-fmk (Figures 2a and b). Under transmission electron micrograph, we randomly selected 100 cells to calculate the percentage of cells undergoing necrosis. Results showed that 57 out of 100 Mz-ChA-1 cells displayed typical necrotic morphology in matrine-treated group, whereas only 13 Mz-ChA-1 cells presented morphologic features of necrosis in Nec-1 plus matrine-treated group (Figure 2c). Similar results were observed in QBC939 cells where 71 and 22% of the 100 cells selected at random underwent necrosis in matrine-treated group and Nec-1 plus matrine-treated group, respectively (Figure 2c). The above data indicated that matrine-induced necroptosis but not apoptosis in CCA cell lines.

### Matrine-induced necroptosis in RIP3-dependent manner

**Figure 3:**
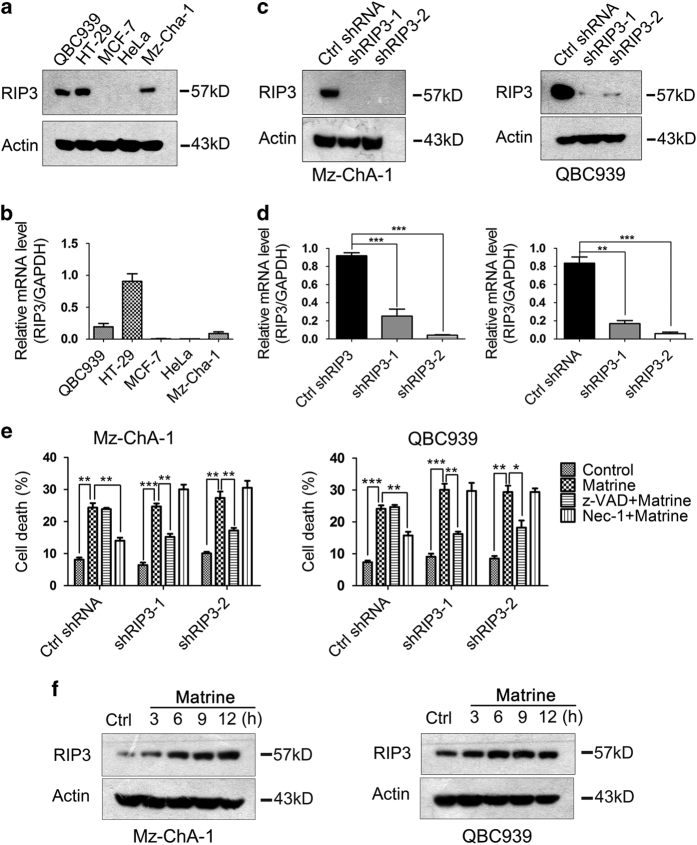
RIP3 was required for matrine to induce necroptosis in CCA cells. (**a** and **b**) Endogenous RIP3 expression levels in several tumor cell lines were detected by western blot (**a**) and real-time PCR (**b**). (**c** and **d**) RIP3 knockdown efficiency in Mz-ChA-1 (Left) and QBC939 (Right) cells was determined by western blot (**c**) and real-time PCR (**d**). *P<0.05, **P<0.01 and ***P<0.001 *versus* control (assessed by Student’s *t*-test). (**e**) Mz-ChA-1 and QBC939 cells expressing control or RIP3 shRNA were pre-treated with Nec-1 (20 *μ*M) or z-VAD-fmk (20 *μ*M) for 2 h, and then treated with matrine (1.5 mg/ml) or vehicle for 48 h. After that, the percentage of cell death was determined by PI staining and flow cytometry. Results were presented as the mean±S.D. from three independent experiments. Significant differences were indicated as *P<0.05, **P<0.01 and ***P<0.001 (assessed by Student’s *t*-test). (**f**) Matrine increased RIP3 expression levels in Mz-ChA-1 and QBC939 cells. Cells were treated with matrine (1.5 mg/ml) for 0, 3, 6, 9 and 12 h, then lysed and subjected to western blot analysis with anti-RIP3 antibody. *β*-actin was used as an internal control.

Receptor-interacting serine-threonine kinase 3 (RIP3) was reported to have a decisive role in necroptosis response.^30^ Its expression was required for cells to undergo necroptosis in response to prototypical necroptosis inducer stimuli TSZ (TNF-*α*+z-VAD-fmk+SMAC mimetic). Therefore, we investigate whether RIP3 was also essential for matrine to induce necroptosis in CCA cells. As RIP3 showed silenced expression in most cancer cells, we first detected the expression levels of RIP3 in Mz-ChA-1 and QBC939 cell lines. HeLa and MCF-7 cells without RIP3 expression and HT-29 cells with high RIP3 expression were used respectively as negative and positive controls. Results from real-time PCR and western blotting showed that RIP3 were highly expressed in QBC939 cells, and moderately expressed in Mz-ChA-1 cells (Figures 3a and b). We further explored the correlation between RIP3 expression and the mode of cell death induced by matrine. Flow cytometry analysis indicated that matrine-induced necroptosis but not apoptosis in QBC939, Mz-ChA-1 and HT-29 cell lines with positive RIP3 expression (Figures 2a and b and Supplementary Figure S1a); in contrast, apoptosis but not necroptosis was induced by matrine in HeLa and MCF-7 cell lines with silenced RIP3 expression (Supplementary Figures S1b and c). These results suggested that the presence of RIP3 protein might switch the cell death from apoptosis to necroptosis when cancer cells were treated with matrine.

We further study the role of RIP3 in matrine-induced cell death. Endogenous RIP3 in Mz-ChA-1 and QBC939 cells was knocked down using lentiviral-mediated RNA interference technology (Figures 3c and d). Result showed that matrine-induced cell death was inhibited by z-VAD-fmk instead of Nec-1 in Mz-ChA-1 and QBC939 cells expressing shRIP3, in opposition to the situation in Mz-ChA-1 and QBC939 cells expressing control shRNA, but similar to that in HeLa and MCF-7 cell lines without RIP3 expression (Figure 3e). These results indicated that matrine-induced necroptosis in RIP3-dependent manner in CCA cells. In addition, knockdown of endogenous RIP3 in HT-29 cells has the similar results in Mz-ChA-1 and QBC939 cells (Supplementary Figures S2a–c). Interestingly, we also found that matrine could increase RIP3 expression levels in Mz-ChA-1 and QBC939 cells (Figure 3f). These results suggested that matrine-induced necroptosis via RIP3 mediation and enhanced RIP3 expression to facilitate the necroptosis.

### MLKL translocation was required in RIP3-mediated necroptosis induced by matrine

**Figure 4:**
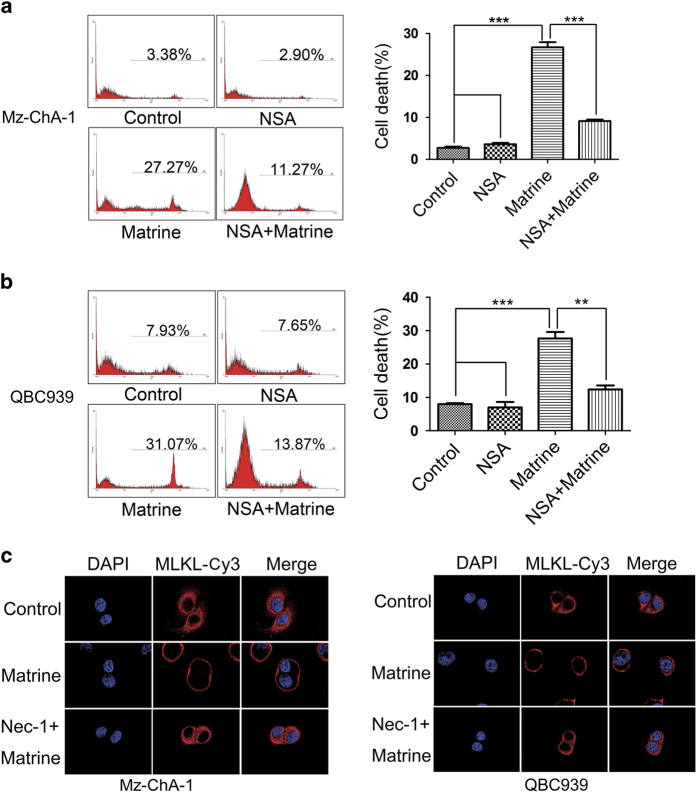
MLKL translocation as a downstream event of RIP3 was required in matrine-induced necroptosis. (**a** and **b**) MLKL was required in matrine-induced necroptosis in Mz-ChA-1 (**a**) and QBC939 (**b**) cells. Cells were pre-treated with MLKL inhibitor NSA (20 nM) for 2 h, and then treated with matrine (1.5 mg/ml) or vehicle for 48 h. After that, the percentage of cell death was determined by PI staining and flow cytometry. Results were presented as the mean±S.D. from three independent experiments. Significant differences were indicated as *P<0.05, **P<0.01 and ***P<0.001 (assessed by Student’s *t*-test). (**c**) MLKL translocation from cytoplasm to plasma membrane induced by matrine were blocked by Nec-1. Mz-ChA-1 and QBC939 cells were pre-treated with Nec-1 (20 *μ*M) for 2 h, and then treated with matrine (1.5 mg/ml) or vehicle for another 2 h. MLKL subcellular localization was analyzed by immunofluorescence and confocal laser scanning microscopy.

We further investigated how RIP3-mediated matrine-induced necroptosis in CCA cells. MLKL is the critical substrate of RIP3 kinase in necroptosis-signaling pathway.^15^ Therefore, we test whether MLKL participated in matrine-induced necroptosis. The results showed that cell death caused by matrine was significantly suppressed by MLKL-specific inhibitor necrosulphonamide in Mz-ChA-1 and QBC939 cells (Figures 4a and b), suggesting that MLKL is involved in matrine-induced cell death. Translocation of MLKL from cytoplasm to cell membrane, leading to membrane rupture, has been proved to be required for TNF*α*-induced necroptosis,^31^ so we next explored whether matrine promoted this translocation of MLKL in CCA cells. Immunofluorescent-staining results showed that MLKL was predominantly located in cytoplasm in intact Mz-ChA-1 and QBC939 cells; whereas most of the MLKL moved to the plasma membrane after matrine treatment, which was prevented by the pretreatment with Nec-1 before matrine (Figure 4c). These data together indicated that MLKL translocation to plasma membrane, as a downstream event of RIP3, was critical for matrine to induce necroptosis.

### ROS generation stimulated by matrine/RIP3/MLKL signaling led to necroptosis

**Figure 5:**
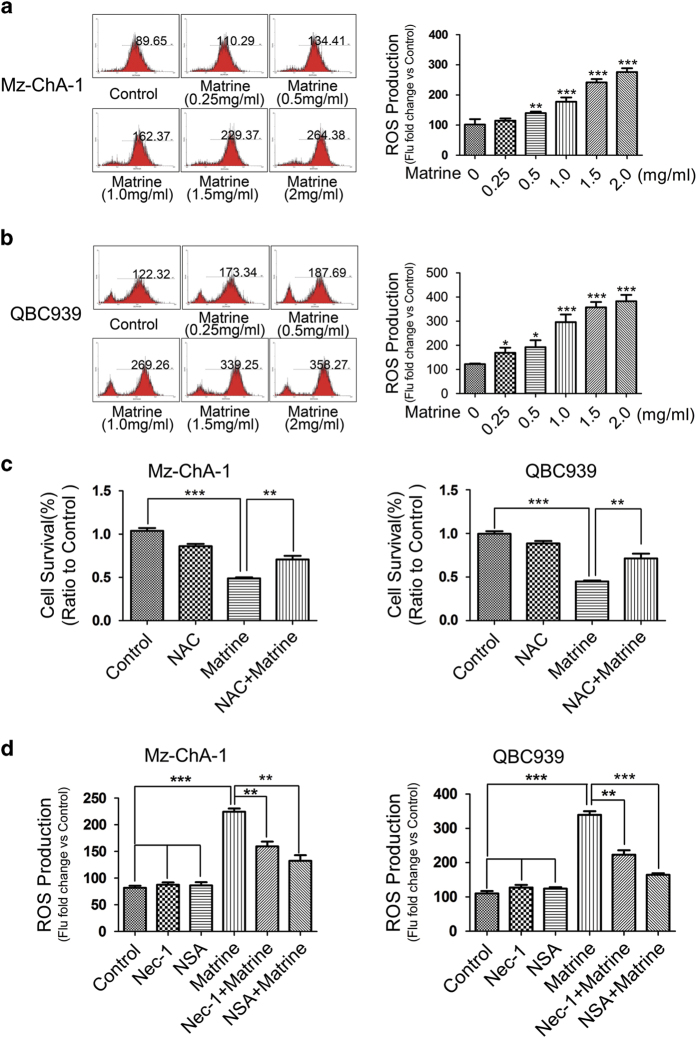
ROS production stimulated by matrine/RIP3/MLKL signaling contributed to matrine-induced necroptosis. (**a** and **b**) Matrine increased the ROS levels of Mz-ChA-1 and QBC939 cells in a dose-dependent manner. Cells were treated with different concentrations of matrine (0, 0.25, 0.5, 1, 1.5 and 2 mg/ml) for 24 h, then the ROS production was measured by flow cytometry. (**c**) Matrine-induced cell death was suppressed by ROS scavenger NAC. Cells were pre-treated with NAC (5 mM) for 3 h, and then treated with matrine (1.5 mg/ml) or vehicle for 48 h. Cell viability was assessed by MTT assay. (**d**) ROS production elevated by matrine were suppressed by Nec-1. Cells were pre-treated with necroptosis inhibitor Nec-1 (20 *μ*M) or NSA (20 nM) for 2 h, and then treated with matrine (1.5 mg/ml) or vehicle for 24 h. ROS levels were detected by flow cytometry. All data were presented as the mean±S.D. from three independent experiments. Significant differences were indicated as *P<0.05, **P<0.01 and ***P<0.001 (assessed by Student’s *t*-test).

It was reported that reactive oxygen species (ROS) production is required for RIP3-mediated necroptosis in several cell lines such as macrophages, MEFs and L929 cells.^30,32–34^ To investigate whether ROS participated in matrine-induced cell death in CCA cells, we first analyzed the effect of matrine on the intracellular ROS levels. Results showed that matrine treatment dose-dependently increased ROS level in both Mz-ChA-1 and QBC939 cell lines (Figures 5a and b). Further study with MTT assay showed that matrine-induced cell death is greatly suppressed by the pretreatment of cells with the ROS scavenger *N*-acetyl-l-cysteine(Figure 5c), suggesting the critical role of ROS production in matrine-induced cell death in QBC939 and Mz-ChA-1 cells. We then analyzed how intracellular ROS levels were elevated by matrine. Results showed that pretreatment of cells with Nec-1 (RIP1 inhibitor) or necrosulphonamide (MLKL inhibitor) could effectively inhibit ROS production (Figure 5d), indicating that ROS production was stimulated by RIP3/MLKL axis. Taken together, these data demonstrated that the activated RIP3/MLKL/ROS signaling pathway also contributed to matrine-induced necroptosis in CCA cells.

### RIP3 was low expressed but not silenced in most CCA tissues

**Figure 6:**
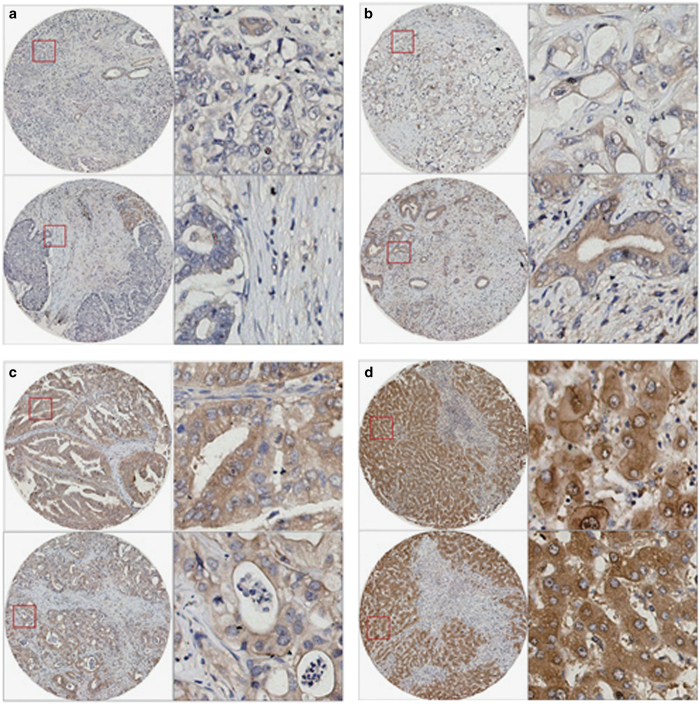
RIP3 was expressed in most CCA tissues. (**a**–**d**) Representative images of IHC using RIP3 antibody in CCA tissues (**a**–**c**) and the paired normal liver tissues (**d**). The left images were shown at ×100 magnification, and the right were corresponding to the left in the red box at ×400 magnification. Yellow represented RIP3, and blue were on behalf of the nucleus. (**a**) Representative images of negative RIP3 expression in CCA tissues (−). (**b**) Representative images of low RIP3 expression in CCA tissues (+). (**c**) Representative images of strong RIP3 expression in CCA tissues (++). (**d**) Representative images of strong RIP3 expression in CCA-adjacent normal liver tissues (++).

Previous reports showed that RIP3 expression is frequently silenced in cancers owing to the methylation of the DNA near RIPK3 transcription start site, which was responsible for the failure of chemotherapeutics via necroptosis induction in cancer cells.^35^ To evaluate the potential of matrine for the treatment of CCA, which required at least low RIP3 expression, we detected the expression levels of RIP3 in CCA tissues and their paired adjacent normal liver tissues. Immunohistochemistry analysis showed that RIP3 protein was mainly located in cytoplasm in CCA tissues and the paired normal liver tissues (Figure 6). RIP3 was expressed in all the detected normal liver tissues (highly expressed in 40 out of the total 42 cases and lowly expressed in the remaining 2 cases) (Figure 6 and Table 1). In contrast, the expression levels of RIP3 in 42 cases of CCA tissues was 13 cases with negative expression (31.0%), 25 cases with low expression (59.5%) and 4 cases with strong expression (9.5%; Figure 6 and Table 1). These results suggested that RIP3 was low expressed but not silenced in most of the CCA tissues.

## Discussion

**Figure 7:**
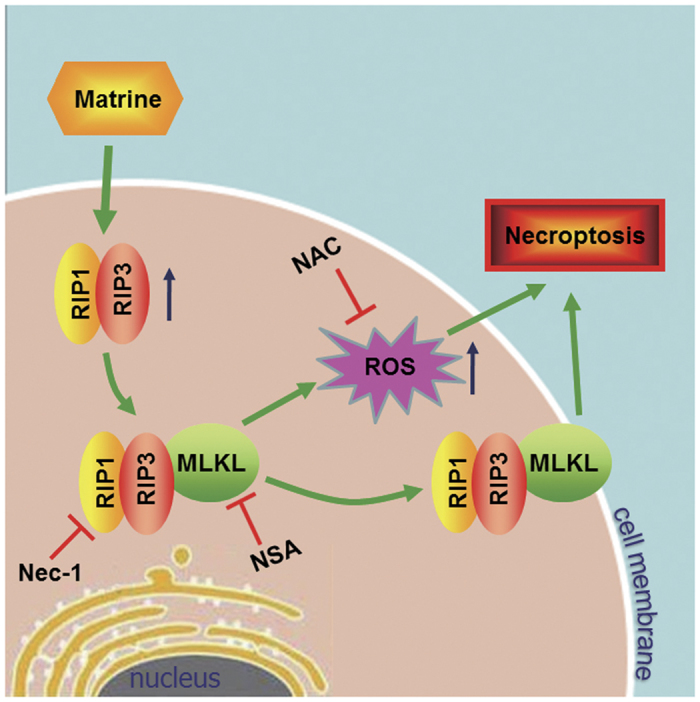
Proposed model for matrine to induce cell necroptosis in CCA cells.

In this study, a novel role of matrine, that is, to induce necroptosis, was discovered in CCA cells and the underlying mechanisms were also investigated (Figure 7). Matrine-induced necroptosis was confirmed by necrotic morphology and the rescue effects of necroptosis inhibitor Nec-1 (Figures 1 and 2). The positive expression of RIP3 was further found to be a molecular switch for matrine to induce necroptosis or apoptosis (Figures 3a–e). Depending on the key function of RIP1/RIP3/MLKL signaling in TNF-induced necroposis,^36^ we proved the upregulated expression of RIP3 (Figure 3f) and plasma membrane translocation of MLKL (Figure 4) in the CCA cell lines under the influence of matrine. What’s more, the increased production of ROS by RIP1/RIP3/MLKL axis was also validated to contribute to matrine-induced necroposis in CCA cell lines (Figure 5). Therefore, two pathways downstream of RIP3 might be involved in matrine-induced necroptosis (Figure 7). The first one, RIP3-activated MLKL translocated to the plasma membrane (Figure 4) and elevated the sodium influx, which led to increased osmotic pressure and ultimately caused membrane rupture and necroptosis.^31^ The other one, activated RIP1/RIP3/MLKL complex increased the generation of ROS (Figure 5), an executioner of necroptosis, via interacting with some metabolic enzymes including glutamate dehydrogenase 1, glutamate-ammonia ligase and glycogen phosphorylase and the mitochondrial protein phosphatase PGAM5.^30,37^ As matrine is generally considered as an apoptosis inductor, which was also supported by our previous experiments on effects of matrine towards Eca-109 and HepG2 cell lines,^38,39^ this necroposis inducing effect of matrine identified in this study will be a promising outbreak in the research of matrine’s cancer-curing mechanisms.

Now some drugs in clinical trials or approved for marketing have been proved as necroptosis inducers to treat different types of cancers, including TRAIL, obatoclax plus dexamethasone, bromopyruvate plus chloroquine and shikonin analogs.^24,40–42^ However, in order to evade from different types of cell death, a lot of cancer cell lines have developed a completed resistance mechanism. After the resistances of apoptosis had been gradually acquainted, anti-chemotherapies cell lines were also observed when treated with necroptosis-inducing drugs. Unlike a relative clear theoretical background of apoptosis-resistance, the report about mechanism of necroptosis-resistance was still very rare. However, what was definite is that RIP3 expression and accumulation is a prerequisite for inducing necroptosis. Numerous cancer cell lines with no RIP3 expression, which make them not sensitive to necroptosis machinery, are unsuitable for necroptosis-based therapy drugs. Recently, studies showed that restoring RIP3 expression could promote those cells’ sensitivity to chemotherapeutics in an RIP3-dependent manner through genomic demethylation near the RIPK3 transcription start site with DNA methylation transferase inhibitor 5-aza-2′-deoxycytidine.^35^ However, 5-aza-2′-deoxycytidine might show great toxic and side effects due to the unwanted demethylation on other DNA regions. Our present study indicated that RIP3 was expressed at low levels in most CCA tumor tissues as compared with normal tissues in CCA patients, which may be the major reason why CCA is insensitive to chemotherapeutic drugs via inducing necroptosis. Exhilaratingly, matrine was found to greatly enhance RIP3 expression in CCA cells, which might solve the problem of chemoresistance in CCA treatment. On the other hand, matrine was unable to induce necroptosis in RIP3-deficient cell lines, which imply a mechanism that matrine’s upregulation of RIP3 is not by demethylation. Of course, the exact mechanism still needs to be further studied.

At present, natural production has been a hot spot in the drug development research to screen targeted cancer therapies. Matrine could meet the two most crucial principles in selecting antitumor drug: efficiency and safety. In clinical therapy, matrine has proved its high efficiency and low toxicity in treating advanced malignant tumors, especially in injection mode.^43–45^ However, an inevitable problem in the application of necroptosis-inducing drugs is their pro-inflammatory effect, which is activated by necroptosis and might exhibit a negative function in tumor treatment. Fortunately, not all necroptosis promote inflammation, sometimes necroptosis process can inhibit inflammatory reactions.^46,47^ Some researchers suggest that induction of necrosis may have the added benefit of invoking the host’s innate immune response to aid cell death and cell necroptosis, which then contribute to immune-surveillance in tumor development.^36,48,49^ Thus, induction of necroptosis in tumors by matrine would be safe for patients.

In conclusion, our study for the first time found that matrine could induce necroptosis in CCA cells with low RIP3 expression by restoring its expression. As a safe clinical drug, matrine might act as a potential effective drug to treat CCA.

## Materials and Methods

### Antibodies and reagents

Matrine (cat.#M5319), *N*-Acetyl-l-cysteine (cat.#A7250), propidium iodide (PI, cat.#P4170) and rabbit anti-MLKL antibody were purchased from Sigma-Aldrich (St Louis, MO, USA). z-VAD-fmk (cat.#627610) and Nec-1 (cat.#480065) were purchased from Merck (KGaA, Darmstadt, Germany). Staurosporine (cat.#1285) was purchased from Tocris Bioscience (Avonmouth, Bristol, UK). Necrosulphonamide (cat.#N388600) was purchased from TRC (Toronto, Ontario, CANADA). DAPI (cat.#10236276001) and DCFH-DA (cat.#KGT010-1) were purchased from KeyGEN (Shanghai, China). DAB Detection Kit (cat.#Kit-0014) was purchased from Fuzhou Maixin Biotech (fuzhou, China). Rabbit anti-RIP3 antibody (cat.#ab72106) and mouse anti-*β*-actin antibody (cat.#ab3280) were purchased from Abcam (Cambridge, MA, USA).

### Cell culture

Human CCA cell lines QBC939 and Mz-ChA-1 were purchased from ATCC, USA. Human cervical cancer cell line HeLa, human colorectal carcinoma cell line HT-29 and human breast cancer cell line MCF-7 were purchased from the Institute of Cell Biology, China. HeLa and MCF-7 Cells were cultured in DMEM (Gibco, Grand Island, NY, USA); QBC939, Mz-ChA-1 and HT-29 cells were cultured in RPMI 1640 (Gibco). All culture media were supplemented with 10% fetal bovine serum (Gibco), 100 U of penicillin, and 100 *μ*g/ml of streptomycin (Life Technologies, Carlsbad, CA, USA). All cells were cultured in a humidified incubator at 37 °C with 5% CO2.

### Cell viability assay

Cellular viability was detected using the MTT method. Cells were cultured in 96-well cell at a density of 8×10^3^ cells per well. After matrine treatment, 20 *μ*l MTT (5 mg/ml) solution was added to the medium directly and incubated for 4 h at 37 °C. Then the medium was removed carefully, and 100 *μ*l DMSO was added to dissolve formazan crystals. The absorbance of each well (OD value) was measured at 570 nm by a Microplate Reader (Bio-Rad, Hercules, CA, USA).

### DAPI staining of the nucleus

Cells were cultured in six-well plates with complete media. After matrine treatment, cells were fixed for 15 min at room temperature using 4% Paraformaldehyde, then washed with phosphate-buffered saline (PBS) twice and stained with DAPI solution (5 *μ*g/ml) for 5 min at 37 °C (protected from light). The samples were observed by fluorescence microscopy. Apoptotic cells were determined by condensed nuclei and formation of apoptotic bodies. The images were chosen from at least three microscopic fields randomly.

### Analysis of cell death by flow cytometry

Cell death was determined by PI exclusion assay. In brief, after matrine treatment, cells were digested using trypsin without EDTA, and collected by centrifugation, then washed twice with PBS and re-suspended in PBS containing 5 *μ*g/ml PI. The tube was gently vortexed and incubated for 5 min at 4 °C in the dark. The samples were analyzed by flow cytometry (Cyflow Space) and the WinMDI (Windows Multiple Document Interface for flow cytometry) software.

### Construction of stable cell lines expressing shRNA

Human RIP3 shRNAs and non-targeting control shRNA were purchased from Shanghai Genechem. The shRNA sequences targeting RIP3 were 5′- GGCTAAACAAACTGAATCT-3′ (shRIP3-1) and 5′- CGACCGCTCGTTAACATAT-3′ (shRIP3-2). The shRNA control (scramble) sequence was 5′- TTCTCCGAACGTGTCACGT-3′. 293T cells were co-transfected with lentiviral-packaging plasmids and control shRNA or RIP3 shRNA plasmid, the virus-containing supernatant was collected 48 h later and used to infect cells. Twelve hours after infection, the cell medium were replaced with fresh complete medium, and puromycin was added 72 h post infection to select stable cell lines.

### Patients, tissue specimens

The study was approved by the Medical Ethics Committee of Zhongshan Hospital Affiliated to Xiamen University and was accorded with the Helsinki Declaration with informed consent of all patients. Samples collected from 42 patients with no preoperative radiation or chemotherapy, one CCA-adjacent normal liver tissue paired with two CCA tissue cores from each patient, in the tissue bank of Zhongshan Hospital Affiliated to Xiamen University.

Tissue microarray was constructed using Formalin-fixed and paraffin-embedded CCA tissues and the matched adjacent normal liver tissues from 42 patients with ALPHELYS MiniCore series 3; 1-mm cores from donor blocks were transferred into a recipient block. The matched normal liver tissues were obtained from a segment of the resected specimens that was >5 cm away from the tumor.

### Immunohistochemistry

Tissue sections were de-waxed by double xylene for 10 min, rehydrated in stepped alcohol, and immersed in 3% hydrogen peroxide for 10 min to suppress endogenous peroxidase activity. Antigen retrieval was performed twice by heating (100 °C) tissue sections for 3 min then cooling for 2 min in 0.01 mol/l sodium citrate buffer (pH 6.0). After three times of 3 min rinses in PBS, tissue sections were incubated for 20 min at room temperature with goat serum. Then, the tissue sections were dried and incubated with the first antibody (anti-RIP3, 1 : 150) incubating at 4 °C overnight. After three washes (each for 3 min in PBS), tissue sections were incubated with biotin-labeled secondary antibody for 10 min at 37 °C. After three additional washes, tissue sections were incubated in streptavidin biotin-peroxidase solution. After three additional washes, peroxidase activity was developed with diaminobenzidine at room temperature, and then counterstained with hematoxylin and washed with water. After routine dehydration and transparency, tissue sections were sealed with neutral resins.

RIP3 protein expression in normal and malignant (CCA) tissues was evaluated by two individuals under fluorescence microscope. Tissue sections were scored per ×40 field. All tissue sections were scored in a semi-quantitative manner, which reflects both the intensity and percentage of cells staining at each intensity. Intensity was classified as 0 (no staining), +1 (weak staining), +2 (distinct staining) or +3 (very strong staining). Percentage was classified as 0 (<10%), +1 (10–25%), +2 (25–50%), +3 (50–75%) and +4 (>75%). A value designated the ‘HSCORE’ was obtained for each slide by using the following algorithm: HSCORE=I×PC, where I and PC represented staining intensity and the percentage of cells, respectively, and the corresponding HSCOREs were calculated separately. RIP3 expressions were classified by HSCORE, negative (0–1), weak positive (2–4), strong positive (4–12). Staining was scored independently by two individuals who were blinded to the findings. *X*^2^-test was used to analyze the results of immunohistochemical staining with the established histopathological malignancy grade.

### Western blot analysis

Cells were lysed in ice-cold RIPA buffer (50 mM Tris-HCl pH 8.0, 150 mM NaCl, 1% NP-40, 0.5% sodium Deoxycholate, 0.1% SDS) containing 1.0 mmol/l phenylmethylsulfonyl fluoride and protease inhibitors (Roche, Indianapolis, IN, USA). After sonication and centrifugation, proteins were separated on 8–10% SDS-polyacrylamide gel and analyzed by immunoblotting. The specific protein bands were visualized by enhanced ECL system (Bio-Rad).

### Real-time PCR

Total RNA was extracted using RNAiso Plus as described by the manufacturer’s protocol (Takara, Dalian, China), and then reverse-transcribed to cDNA using Primescript RT reagent kit (TaKaRa). Real-Time PCR was performed using the SYBR Green I fluorescent dye (SYBR Premix Ex Taq II, TaKaRa) and the StepOnePlus real-time PCR system (Applied Biosystems, Australia). The PCR condition is the following: an initial pre-degeneration at 95 °C for 2 min, followed by 40 cycles of denaturation at 95 °C for 10 s and annealing/extension at 60 °C for 20 s. The fold change in mRNA expression level was calculated using the comparative ΔCt method and GAPDH as a normalization control. The primers used were listed below:

*RIP3*, 5′- ACTCCCGGCTTAGAAGGACT-3′ (forward)

5′- GCCCTGCTCCTCTTGGTAAG-3′ (reverse)

*GAPDH*, 5′- TGCACCACCAACTGCTTAGC-3′ (forward)

5′- GGCATGGACTGTGGTCATGAG-3′ (reverse)

### ROS detection

Cells were washed three times with PBS and stained with 20 *μ*M DCFH-DA for 30 min in 37 °C, 5% CO_2_ incubator. Then the cells were trypsinized, collected by centrifugation, washed again using PBS, and re-suspended in 1 ml PBS. ROS generation was measured by the flow cytometry (Cyflow Space, Partec, Germany) and the WinMDI (Windows Multiple Document Interface for flow cytometry) software.

### Immunofluorescence

Cells were seeded on coverslips in six-well plates with complete media. After matrine treatment, cells were washed three times with PBS and fixed with 4% paraformaldehyde for 15 min at room temperature. The fixed cells were washed three times and then permeabilized in 0.3% Triton X-100 (in 0.02% BSA/PBS) for 20 min. Next, the cells were blocked with 5% BSA in PBS for 30 min and washed three times with PBS, and then stained with anti-MLKL antibody (rabbit, 1:100, Sigma) for 3 h at room temperature. The cells were then washed three times in PBS followed by incubating with the FITC-labeled anti-rabbit second antibody (1:200, Santa Cruz Biotech, CA, USA) 1 h in dark environment. Cells were washed three times with PBS and stained with DAPI for 5 min. At last, slides were mounted with fluoromount. Samples were analyzed by the confocal laser scanning microscope. The confocal images results were representative of at least three independent experiments.

### Statistical analysis

The above experiments were repeated for at least three times. The data were analyzed by prism 5.0 (GraphPad, San Diego, CA, USA) and expressed as the mean±S.D., and the significant difference was determined by the student’s *t*-test. *P*<0.05 was considered as significant.

## Figures and Tables

**Table 1:** RIP3 expression compared in CCA tissues and their paired adjacent normal liver tissues

*Clinical parameters*	*Number*	*RIP3 expression*			*Positive rate*	P *value*
		*(−)*	*(+)*	*(++)*		
Normal liver tissues	42	0	2	40	100%	<0.05
CCA tissues	42	13	25	4	69.04%	

## References

[bib1] Sandhu DS, Roberts LR. Diagnosis and management of cholangiocarcinoma. Curr Gastroenterol Rep 2008; 10: 43–52. 18417042 10.1007/s11894-008-0008-9

[bib2] Soares KC, Kamel I, Cosgrove DP, Herman JM, Pawlik TM. Hilar cholangiocarcinoma: diagnosis, treatment options, and management. Hepatobil Surg Nutr 2014; 3: 18–34. 10.3978/j.issn.2304-3881.2014.02.05 PMC3955000 24696835

[bib3] Fabris L, Alvaro D. The prognosis of perihilar cholangiocarcinoma after radical treatments. Hepatology 2012; 56: 800–802. 22532318 10.1002/hep.25808

[bib4] Olnes MJ, Erlich R. A review and update on cholangiocarcinoma. Oncology 2004; 66: 167–179. 15218306 10.1159/000077991

[bib5] Goenka MK, Goenka U. Palliation: hilar cholangiocarcinoma. World J Hepatol 2014; 6: 559–569. 25232449 10.4254/wjh.v6.i8.559 PMC4163739

[bib6] Teng X, Degterev A, Jagtap P, Xing X, Choi S, Denu R et al. Structure-activity relationship study of novel necroptosis inhibitors. Bioorg Med Chem Lett 2005; 15: 5039–5044. 16153840 10.1016/j.bmcl.2005.07.077

[bib7] Tait SW, Green DR. Caspase-independent cell death: leaving the set without the final cut. Oncogene 2008; 27: 6452–6461. 18955972 10.1038/onc.2008.311 PMC2635930

[bib8] Bonnet MC, Preukschat D, Welz PS, van Loo G, Ermolaeva MA, Bloch W et al. The adaptor protein FADD protects epidermal keratinocytes from necroptosis in vivo and prevents skin inflammation. Immunity 2011; 35: 572–582. 22000287 10.1016/j.immuni.2011.08.014

[bib9] Welz PS, Wullaert A, Vlantis K, Kondylis V, Fernandez-Majada V, Ermolaeva M et al. FADD prevents RIP3-mediated epithelial cell necrosis and chronic intestinal inflammation. Nature 2011; 477: 330–334. 21804564 10.1038/nature10273

[bib10] Vanlangenakker N, Berghe TV, Vandenabeele P. Many stimuli pull the necrotic trigger, an overview. Cell Death Differ 2012; 19: 75–86. 22075985 10.1038/cdd.2011.164 PMC3252835

[bib11] Zhang DW, Shao J, Lin J, Zhang N, Lu BJ, Lin SC et al. RIP3, an energy metabolism regulator that switches TNF-induced cell death from apoptosis to necrosis. Science 2009; 325: 332–336. 19498109 10.1126/science.1172308

[bib12] Koo G-B, Morgan MJ, Lee D-G, Kim W-J, Yoon J-H, Koo JS et al. Methylation-dependent loss of RIP3 expression in cancer represses programmed necrosis in response to chemotherapeutics. Cell Res 2015; 25: 707–725. 25952668 10.1038/cr.2015.56 PMC4456623

[bib13] Moriwaki K, Bertin J, Gough P, Orlowski G, Chan FK. Differential roles of RIPK1 and RIPK3 in TNF-induced necroptosis and chemotherapeutic agent-induced cell death. Cell Death Dis 2015; 6: e1636. 25675296 10.1038/cddis.2015.16 PMC4669795

[bib14] Nugues A-L, El Bouazzati H, Hetuin D, Berthon C, Loyens A, Bertrand E et al. RIP3 is downregulated in human myeloid leukemia cells and modulates apoptosis and caspase-mediated p65/RelA cleavage. Cell Death Dis 2014; 5: e1384. 25144719 10.1038/cddis.2014.347 PMC4454320

[bib15] Sun L, Wang H, Wang Z, He S, She C, Liao D et al. Mixed lineage kinase domain-like protein mediates necrosis signaling downstream of RIP3 kinase. Cell 2012; 148: 213–227. 22265413 10.1016/j.cell.2011.11.031

[bib16] Galluzzi L, Vanden BT, Vanlangenakker N, Buettner S, Eisenberg T, Vandenabeele P et al. Programmed necrosis from molecules to health and disease. Int Rev Cell Mol Biol 2011; 289: 1–35. 21749897 10.1016/B978-0-12-386039-2.00001-8

[bib17] Han J, Zhong CQ, Zhang DW. Programmed necrosis: backup to and competitor with apoptosis in the immune system. Nat Immunol 2011; 12: 1143–1149. 22089220 10.1038/ni.2159

[bib18] Sun L, Wang H, Wang Z, He S, Chen S, Liao D et al. Mixed lineage kinase domain-like protein mediates necrosis signaling downstream of RIP3 kinase. Cell 2012; 148: 213–227. 22265413 10.1016/j.cell.2011.11.031

[bib19] Cai Z, Jitkaew S, Zhao J, Chiang HC, Choksi S, Liu J et al. Plasma membrane translocation of trimerized MLKL protein is required for TNF-induced necroptosis. Nat Cell Biol 2014; 16: 55–65. 24316671 10.1038/ncb2883 PMC8369836

[bib20] Ma LD, Wen SH, Zhan Y, He YJ, Uu XS, Jiang JK. Anticancer effects of the chinese medicine matrine on murine hepatocellular carcinoma cells. Planta Med 2008; 74: 245–251. 18283616 10.1055/s-2008-1034304

[bib21] Xin HB, Liu SF. [Effects of matrine on myocardial contraction and arrhythmia in isolated heart atria]. Zhongguo yao li xue bao 1987; 8: 501–505. 3451660

[bib22] Liu TY, Song Y, Chen H, Pan SH, Sun XY. Matrine inhibits proliferation and induces apoptosis of pancreatic cancer cells in vitro and in vivo. Biol Pharm Bull 2010; 33: 1740–1745. 20930385 10.1248/bpb.33.1740

[bib23] Zhang S, Zhang Y, Zhuang Y, Wang J, Ye J, Zhang S et al. Matrine induces apoptosis in human acute myeloid leukemia cells via the mitochondrial pathway and Akt inactivation. Plos One 2012; 7: e46853. 23056487 10.1371/journal.pone.0046853 PMC3466205

[bib24] Zhou H, Xu M, Gao Y, Deng Z, Cao H, Zhang W et al. Matrine induces caspase-independent program cell death in hepatocellular carcinoma through bid-mediated nuclear translocation of apoptosis inducing factor. Molecular Cancer 2014; 13: 1–11. 24628719 10.1186/1476-4598-13-59 PMC4007561

[bib25] He W, Wang Q, Srinivasan B, Xu J, Padilla MT, Li Z et al. A JNK-mediated autophagy pathway that triggers c-IAP degradation and necroptosis for anticancer chemotherapy. Oncogene 2014; 33: 3004–3013. 23831571 10.1038/onc.2013.256 PMC3912228

[bib26] Yu X, Deng Q, Li W, Xiao L, Luo X, Liu X et al. Neoalbaconol induces cell death through necroptosis by regulating RIPK-dependent autocrine TNF*α* and ROS production. Oncotarget 2015; 6: 1995–2008. 25575821 10.18632/oncotarget.3038 PMC4385831

[bib27] Hu J, Liu X, Hughes D, Esteva FJ, Liu B, Chandra J, Li S. Herceptin conjugates linked by EDC boost direct tumor cell death via programmed tumor cell necrosis. Plos One 2011; 6: e23270. 21853100 10.1371/journal.pone.0023270 PMC3154407

[bib28] Coupienne I, Fettweis G, Rubio N, Agostinis P, Piette J. 5-ALA-PDT induces RIP3-dependent necrosis in glioblastoma. Photochem Photobiol Sci 2011; 10: 1868–1878. 22033613 10.1039/c1pp05213f

[bib29] Han W, Ling L, Shuang Q, Lu Q, Pan Q, Ying G et al. Shikonin circumvents cancer drug resistance by induction of a necroptotic death. Mol Cancer Ther 2007; 6: 1641–1649. 17513612 10.1158/1535-7163.MCT-06-0511

[bib30] Zhang DW, Shao J, Lin J, Zhang N, Lu BJ, Lin SC et al. RIP3, an energy metabolism regulator that switches TNF-induced cell death from apoptosis to necrosis. Science 2009; 325: 332–336. 19498109 10.1126/science.1172308

[bib31] Chen X, Li W, Ren J, Huang D, He WT, Song Y et al. Translocation of mixed lineage kinase domain-like protein to plasma membrane leads to necrotic cell death. Cell Res 2013; 24: 105–121. 24366341 10.1038/cr.2013.171 PMC3879712

[bib32] He S, Wang L, Miao L, Wang T, Du F, Zhao L et al. Receptor interacting protein kinase-3 determines cellular necrotic response to TNF-*α*. Cell 2009; 137: 1100–1111. 19524512 10.1016/j.cell.2009.05.021

[bib33] Vanlangenakker N, Vanden BT, Bogaert P, Laukens B, Zobel K, Deshayes K et al. cIAP1 and TAK1 protect cells from TNF-induced necrosis by preventing RIP1/RIP3-dependent reactive oxygen species production. Cell Death Differ 2011; 18: 656–665. 21052097 10.1038/cdd.2010.138 PMC3131911

[bib34] Kim SO, Ono K, Tobias PS, Han J. Orphan nuclear receptor Nur77 is involved in caspase-independent macrophage cell death. J Exp Med 2003; 197: 1441–1452. 12782711 10.1084/jem.20021842 PMC2193909

[bib35] Koo GB, Morgan MJ, Lee DG, Kim WJ, Yoon JH, Koo JS et al. Methylation-dependent loss of RIP3 expression in cancer represses programmed necrosis in response to chemotherapeutics. Cell Res 2015; 25: 707–725. 25952668 10.1038/cr.2015.56 PMC4456623

[bib36] Han J, Zhong CQ, Zhang DW. Programmed necrosis: backup to and competitor with apoptosis in the immune system. Nat Immunol 2011; 12: 1143–1149. 22089220 10.1038/ni.2159

[bib37] Wang Z, Hui J, She C, Du F, Wang X. The mitochondrial phosphatase PGAM5 functions at the convergence point of multiple necrotic death pathways. Cell 2012; 148: 228–243. 22265414 10.1016/j.cell.2011.11.030

[bib38] Huan Z, Ming-ying X, Yu L, Kai L, Qing G, Tian-hui H et al. Matrine induces apoptosis of Hep G2 cells via mitochondrial apoptotic pathway. Guangxi Medical Journal 2014; 11: 1588–1592.

[bib39] Wang Q, Du H, Geng G, Zhou H, Xu M, Cao H et al. Matrine inhibits proliferation and induces apoptosis via BID-mediated mitochondrial pathway in esophageal cancer cells. Mol Biol Rep 2014; 41: 3009–3020. 24510386 10.1007/s11033-014-3160-3

[bib40] Meurette O, Rebillard A, Huc L, Le MG, Merino D, Micheau O et al. TRAIL induces receptor-interacting protein 1-dependent and caspase-dependent necrosis-like cell death under acidic extracellular conditions. Cancer Res 2007; 67: 218–226. 17210702 10.1158/0008-5472.CAN-06-1610

[bib41] Xuan Y, Hu X. Naturally-occurring shikonin analogues–a class of necroptotic inducers that circumvent cancer drug resistance. Cancer Lett 2009; 274: 233–242. 19027226 10.1016/j.canlet.2008.09.029

[bib42] Bonapace L, Bornhauser BC, Schmitz M, Cario G, Ziegler U, Niggli FK et al. Induction of autophagy-dependent necroptosis is required for childhood acute lymphoblastic leukemia cells to overcome glucocorticoid resistance. J Clin Invest 2010; 120: 1310–1323. 20200450 10.1172/JCI39987 PMC2846044

[bib43] Ma X, Li RS, Wang J, Huang YQ, Li PY, Wang J et al. The therapeutic efficacy and safety of compound kushen injection combined with transarterial chemoembolization in unresectable hepatocellular carcinoma: an update systematic review and meta-analysis. Front Pharmacol 2016; 7: 70. 27065861 10.3389/fphar.2016.00070 PMC4814457

[bib44] Yan LI, Cao WW, Yang YL, Zou H, Jiang XT. Matrine sustained release tablet, capsule and injection: comparison of pharmacokinetics and relative bioavailability. Acad J Second Mil Med Univ 2005; 6: 681–683.

[bib45] Lao Y. Clinicalstudy of matrine injection on preventing liver function damage of anti-tumor drugs during chemotherapy of breast cancer. J Chin Med Mater 2005; 28: 735–737. 16379429

[bib46] Casares N, Pequignot MO, Tesniere A, Ghiringhelli F, Roux S, Chaput N et al. Caspase-dependent immunogenicity of doxorubicin-induced tumor cell death. J Exp Med 2005; 202: 1691–1701. 16365148 10.1084/jem.20050915 PMC2212968

[bib47] Hirt UA, Leist M. Rapid, noninflammatory and PS-dependent phagocytic clearance of necrotic cells. Cell Death Differ 2003; 10: 1156–1164. 14502239 10.1038/sj.cdd.4401286

[bib48] Gamrekelashvili J, Krüger C, Wasielewski RV, Hoffmann M, Huster KM, Busch DH et al. Necrotic tumor cell death in vivo impairs tumor-specific immune responses. J Immunology 2007; 178: 1573–1580. 17237406 10.4049/jimmunol.178.3.1573

[bib49] Young LS, Rickinson AB. Epstein-Barr virus: 40 years on. Nat Rev Cancer 2004; 4: 757–768. 15510157 10.1038/nrc1452

