# Supplementary figures and images for: Matrine induces RIP3-dependent necroptosis in cholangiocarcinoma cells
Source: Cell Death Discov. 2017 Jan 23;3:16096–. doi: 10.1038/cddiscovery.2016.96 (PMC5253620; doi:10.1038/cddiscovery.2016.96)

# Figure S1

a

HT-29

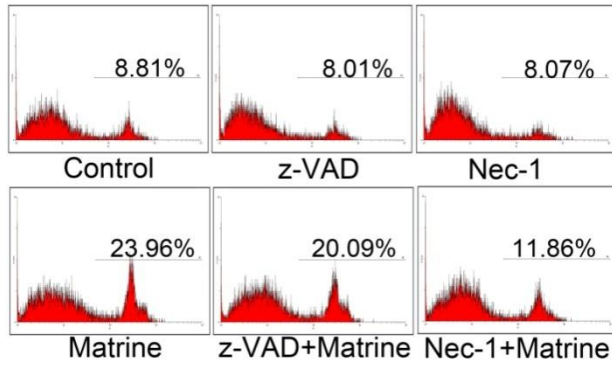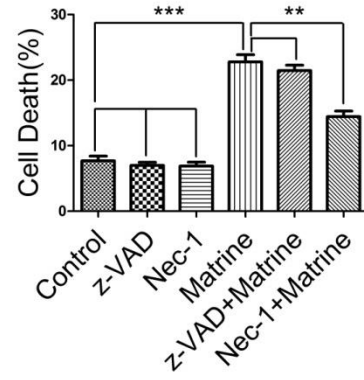

b

HeLa

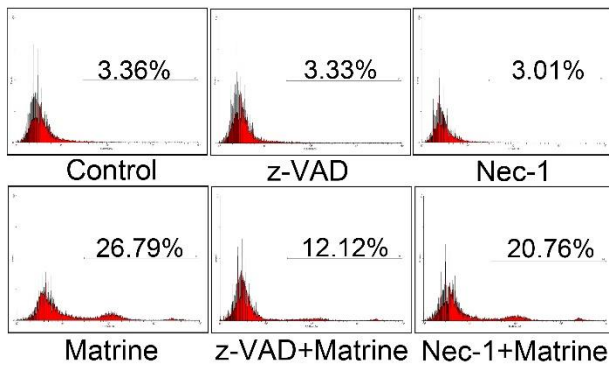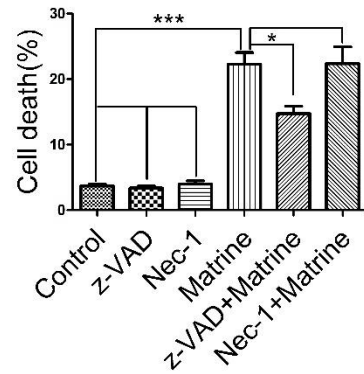

c

MCF-7

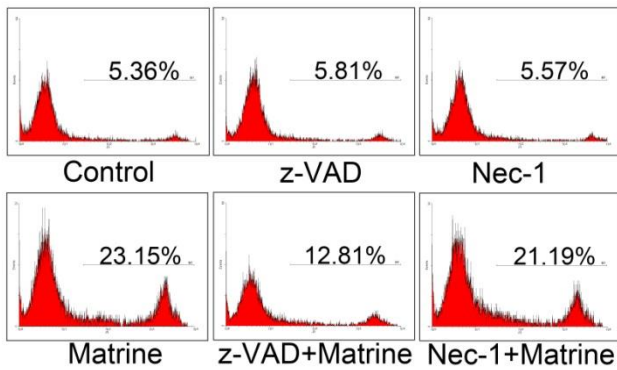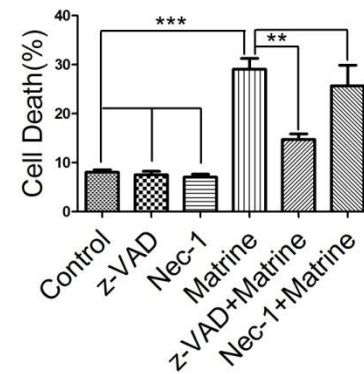

Figure S2

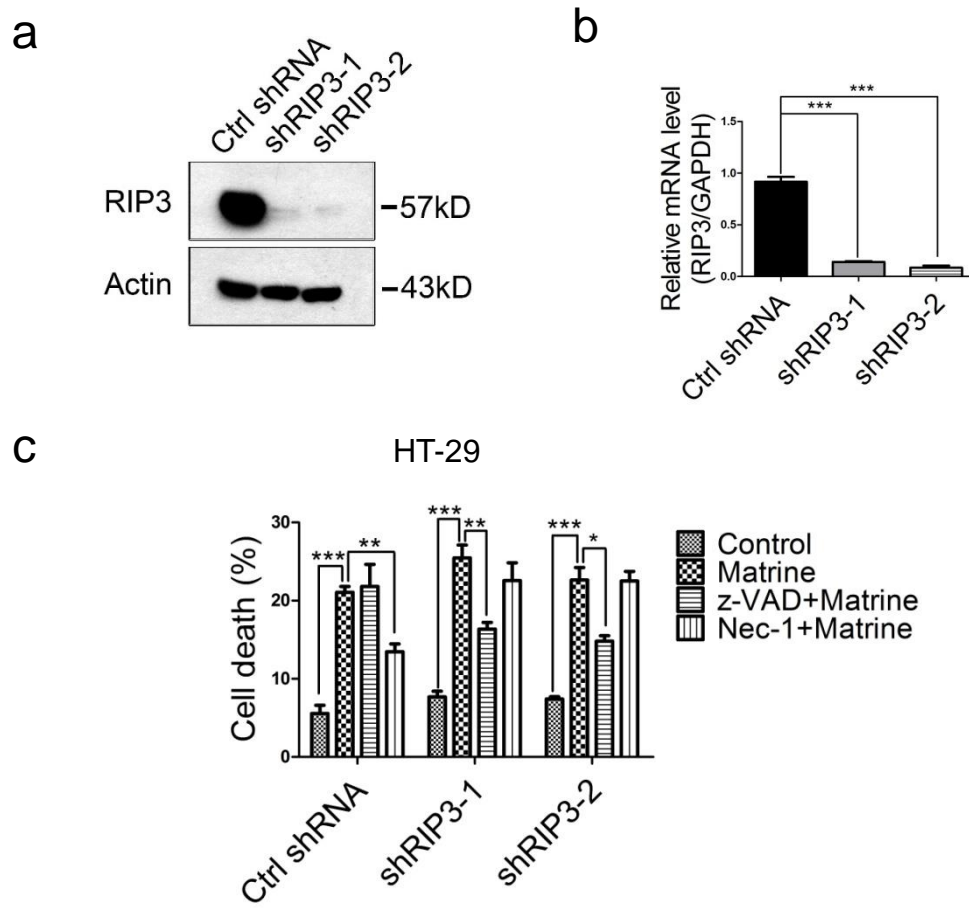

Supplement: Supplementary Figures [file cddiscovery201696-s1.pdf]
