# Supplementary material for: Matrine induces RIP3-dependent necroptosis in cholangiocarcinoma cells
Source: Cell Death Discov. 2017 Jan 23;3:16096–. doi: 10.1038/cddiscovery.2016.96 (PMC5253620; doi:10.1038/cddiscovery.2016.96)
Supplement: Supplementary Figure Legends [file cddiscovery201696-s2.doc]

**Figure S1. Matrine induced different mode of cell death in HT-29, HeLa and MCF-7 cell lines.**

(a-c) HT-29, HeLa and MCF-7 cells were pre-treated with necroptosis inhibitor Nec-1 (20 μM) or caspase dependent apoptosis inhibitor z-VAD-fmk (20 μM) for 2 h, and then treated with matrine (1.5 mg/ml) or vehicle for 48 h. After that, the percentage of cell death was determined by PI staining and flow cytometry. Results were presented as the mean ± SD from three independent experiments. Significant differences were indicated as *p<0.05, **p<0.01 and ***p<0.001 (assessed by Student’s *t*-test).

**Figure S2. RIP3 is required for matrine to induce necroptosis in HT-29 cells**

(a-b) RIP3 knockdown efficiency in HT-29 cells was determined by western blot (a) and real-time PCR (b). * *p*< 0.05, ***p*<0.01, and ****p*<0.001 *vs* control (assessed by Student’s t-test). (c) HT-29 cells expressing control or RIP3 shRNA were pre-treated with Nec-1 (20 μM) or z-VAD-fmk (20 μM) for 2 h, and then treated with matrine (1.5 mg/ml) or vehicle for 48 h. After that, the percentage of cell death was determined by PI staining and flow cytometry. Results were presented as the mean ± SD from three independent experiments. Significant differences were indicated as **p*<0.05, ***p*<0.01 and ****p*<0.001 (assessed by Student’s t-test).
